# Integrating comprehensive geriatric assessment into routine nursing care for older adults with type 2 diabetes: implementation fidelity and clinical outcomes

**DOI:** 10.3389/fpubh.2025.1665732

**Published:** 2025-09-19

**Authors:** Qinqin Sun, Dongmei Ren, Jianping Tong, Li Ao, Shaowen Wang

**Affiliations:** ^1^Department of Nursing, Shanghai Jiading District Central Hospital, Shanghai, China; ^2^Department of Nursing, Shanghai Songjiang District Si Jing Hospital, Shanghai, China

**Keywords:** implementation fidelity, comprehensive geriatric assessment, type 2 diabetes, older adults, nurse-led care, healthcare utilization

## Abstract

**Background:**

Comprehensive geriatric assessment (CGA) offers promise for improving diabetes management in older adults; however, its real-world effectiveness depends on implementation fidelity, which remains poorly understood. This study examined fidelity variations and their associations with clinical outcomes in nurse-led CGA for older adults with type 2 diabetes at a tertiary care hospital in China.

**Methods:**

This cross-sectional implementation study enrolled 3,351 adults aged ≥65 years with type 2 diabetes from Shanghai Jiading District Central Hospital between March 2021 and February 2025. Implementation fidelity was assessed using five validated dimensions yielding a composite score (mean 0.64, SD 0.19; range 0.28–0.94). Primary outcome was glycated hemoglobin (HbA1c); secondary outcomes included cardiometabolic parameters, patient-centered measures, healthcare utilization, and hypoglycemic events. Linear regression models with robust standard errors adjusted for confounders; mediation analysis examined functional status pathways.

**Results:**

Fidelity demonstrated variation within the hospital (mean 0.64, SD 0.19; range 0.28–0.94), with higher educational attainment, provider experience, and CGA training completion associated with better implementation quality. Higher fidelity was associated with lower HbA1c (adjusted β −0.38 per 0.10-unit increase, 95% CI −0.47 to −0.29; *p* < 0.001), with a graded association across quartiles [7.89% (95% CI 7.78–8.00) in the lowest quartile vs. 7.16% (95% CI 7.04–7.28) in the highest quartile; *p* for trend < 0.001]. Benefits were associated with lower systolic blood pressure (−5.10 mm Hg, 95% CI −7.20 to −3.00), LDL cholesterol (−6.50 mg/dl, 95% CI −9.10 to −3.90), improved quality of life (EuroQol-5D: 0.061, 95% CI 0.041–0.081), and decreased depressive symptoms (−1.10, 95% CI −1.40 to −0.80; all *p* < 0.001). Healthcare utilization declined (hospitalization incidence rate ratio 0.61, 95% CI 0.51–0.73; *p* < 0.001), and odds of hypoglycemic events were lower (odds ratio 0.78, 95% CI 0.72–0.84; *p* < 0.001). Functional status was an estimated mediator of 31.6% of the fidelity–HbA1c association (indirect β −0.12, 95% CI −0.17 to −0.07; *p* < 0.001), with age and gait speed modifying associations (*p* = 0.04 and 0.02, respectively).

**Conclusion:**

High-fidelity CGA integration is associated with substantial clinical benefits and lower healthcare utilization; while suggestive of economic advantages, a formal cost-effectiveness evaluation was not undertaken. These associations support an institutional focus on provider training, experience development, and patient education to optimize geriatric diabetes care quality.

## 1 Introduction

The rising prevalence of type 2 diabetes mellitus in older adults presents a significant public health concern, intensified by global population aging. In the United States, 26% of adults aged ≥65 years have diagnosed diabetes, and an additional 50% exhibit prediabetes, contributing to elevated morbidity, mortality, and healthcare costs (1). China, home to the world's largest diabetic population, reports prevalence rates of 23.9% in individuals aged 60–69 and 27.3% in those ≥70 years, affecting approximately 74 million older adults among its 269 million citizens aged ≥60. By 2045, global diabetes cases in adults ≥65 are projected to surpass 276 million, with China disproportionately affected due to urbanization and lifestyle transitions (2, 3). These trends are compounded by age-related declines in insulin sensitivity and beta-cell function, heightening risks of glycemic instability and complications such as cardiovascular disease and neuropathy (4–6). Thus, innovative care models must integrate metabolic management with geriatric considerations to mitigate adverse outcomes and enhance resource efficiency (7).

In China, the rapid expansion of the older adult population, combined with the increasing prevalence of type 2 diabetes, poses significant challenges for healthcare delivery. National guidelines underscore the urgency of addressing chronic diseases among older adults patients, and diabetes management in this group is particularly complex due to the frequent coexistence of hypertension, cardiovascular disease, and other comorbid conditions (8). The multifaceted nature of these challenges mandates a shift from conventional disease-specific management toward a more holistic approach that considers the physical, psychological, and social dimensions of health. Comprehensive geriatric assessment (CGA) represents a promising strategy to meet these demands by facilitating a multidimensional evaluation that is crucial for tailoring individualized care plans (9, 10).

While CGA has gained ground internationally to improve health outcomes among older adults, its routine integration into nursing care within China remains in its infancy. Recent studies indicate that CGA was introduced in China primarily in outpatient and community settings, but its application in inpatient environments—especially among older patients with diabetes—is still limited (9, 10). This gap is compounded by challenges such as inadequate training of nursing staff, insufficient knowledge about geriatric care competencies, and varying levels of familiarity with CGA methodologies (10, 11). Moreover, given the escalating demand for specialized nursing services driven by demographic changes, there is an urgent need to advance nurse training and adopt systematic approaches for the integration of CGA into everyday clinical practice (10, 12, 13).

In China, nurses occupy a central role in the management of diabetes among older adults, frequently serving as primary caregivers and playing a vital role in patient education and care coordination (14). Incorporating CGA into routine nursing practice promises to enhance the quality of care by identifying functional impairments, psychosocial issues, and geriatric syndromes that are frequently overlooked in standard clinical assessments (9, 10). This integration may improve care continuity, decrease the likelihood of adverse outcomes such as hospital readmissions, and strengthen the overall clinical effectiveness of diabetes management (9). Nonetheless, despite its potential, there remains a critical gap in empirical evidence assessing the fidelity of CGA implementation in routine nursing care and its associated clinical outcomes within the Chinese healthcare context.

Therefore, the current study investigated the implementation fidelity of integrating comprehensive geriatric assessment into routine nursing care for older adults with type 2 diabetes in a healthcare facility in China, and examined its association with glycemic control, cardiometabolic outcomes, functional status, and healthcare utilization. Key objectives included quantifying fidelity using validated composite scores, identifying patient- and provider-level predictors, exploring mediation pathways via functional measures, and conducting sensitivity analyses to test effect robustness. The findings aim to guide scalable models for optimizing geriatric diabetes care in clinical settings.

## 2 Methodology

This cross-sectional implementation research study incorporated retrospective outcome data collection to evaluate the integration of CGA into routine nursing care for older adults with type 2 diabetes mellitus. Conducted March 2021–February 2025 at Shanghai Jiading District Central Hospital in China, the investigation utilized quantitative evaluations of implementation fidelity and clinical outcomes to elucidate the association between intervention delivery quality and patient health status. Furthermore, the design addressed gaps in implementation science by identifying modifiable factors influencing CGA effectiveness in a single-center real-world setting, thereby informing strategies for optimizing geriatric diabetes care. This approach was particularly suited to examining variations in implementation quality among patients and providers, with a focus on how fidelity modulates clinical impacts in heterogeneous patient populations. Retrospective elements included 12-month reviews of healthcare utilization and adverse events, which supplemented cross-sectional assessments to provide temporal context without implying longitudinal follow-up. To minimize concerns of reverse causation, all index CGA encounters occurred ≥3 months before laboratory sampling for HbA1c and other outcomes, ensuring fidelity assessments preceded outcome ascertainment.

### 2.1 Setting and participants

The study was conducted at Shanghai Jiading District Central Hospital, a tertiary healthcare facility within China's tiered system. The hospital had adopted standardized CGA protocols within the prior 24 months under a national initiative, allowing assessment of implementation variations among different providers and patient populations. Participants systematically sampled using consecutive enrollment during routine visits, included adults aged ≥65 years with type 2 diabetes duration ≥6 months, regular hospital attendance, informed consent capability, and ≥1 CGA session in the past 12 months. Exclusions comprised severe cognitive impairment (mini-mental state examination < 10), terminal illness (life expectancy < 6 months), temporary residence (< 6 months), or assessment-incompatible physical limitations. The final cohort totaled 3,351 participants enrolled across four annual periods from March 2021 to February 2025. To address potential selection bias, participant demographics were examined across enrollment periods. Sample size was calculated to detect a beta coefficient of −0.40 for HbA1c (%) per 0.10-unit increase in fidelity score (SD = 1.2% for HbA1c, SD = 0.19 for fidelity), assuming 80% power, α = 0.05. For a single-center design, this yielded a requirement of approximately 400 participants; the achieved 3,351 provided substantial power for primary analyses and robust subgroup examinations. A *post-hoc* calculation confirmed >80% power for detecting the targeted effect size, though necessitating cautious interpretation of subgroup and mediation results with lower precision.

### 2.2 Implementation of comprehensive geriatric assessment

The CGA protocol, developed by geriatric and diabetes experts, spanned five domains: functional assessment (activities of daily living, instrumental activities of daily living, mobility); cognitive evaluation (memory, executive function); mood screening (depression); social circumstances (support systems, determinants of health); and diabetes-specific planning (medication, self-care, hypoglycemia risk, goals). Nurses completed assessments in 45–60 min, generating tailored care plans with standardized documentation. Nursing staff received 16-h initial training, workflow adaptation sessions, monthly supervision, and utilized electronic decision support systems. These elements ensured protocol feasibility while accommodating individual patient needs and variations in provider experience that could influence fidelity. Fidelity assessments were conducted concurrently with outcome data collection during the study period, with implementation quality evaluated based on CGA sessions occurring within the preceding 12 months to align temporally with clinical measures.

### 2.3 Fidelity assessment

Implementation fidelity was evaluated across five dimensions: adherence (protocol completion by nurses); dose (session frequency/duration delivered by nurses); quality of delivery (provider competency); participant responsiveness (patient engagement); and program differentiation (distinction from usual care, reverse-scored). Nurse performance was assessed through direct observations by trained research staff (20% random sample), medical record abstraction by independent reviewers, and self-report questionnaires. Patient engagement was measured via satisfaction surveys administered by research assistants and engagement metrics from CGA documentation. Program distinctiveness was evaluated through administrator interviews and comparison with standard care protocols. Evaluation targets were defined *a priori*: adherence, dose, and quality of delivery were assessed at the provider–encounter level; participant responsiveness at the patient level; and program differentiation at the unit level.

Test-retest reliability was established via dual coding (20% observations), yielding reliability coefficients of 0.88–0.94. Scoring used validated scales: adherence as proportion completed; dose on a 6-point scale; quality and responsiveness on 5-point scales; differentiation binary. The composite fidelity score was derived as a weighted average of standardized domain scores, with weights obtained from principal component analysis conducted on the full sample; the first principal component explained 64.8% of variance (Table 1), and weights were proportional to loadings (e.g., adherence: 0.82, dose: 0.79). To mitigate overfitting, weights were derived on a random 70% subsample and validated on the remaining 30%, with consistent loadings (differences < 0.05); additional sensitivities imposed equal weighting across domains and excluded self-reported items, confirming robustness of primary associations. This multi-method approach minimized measurement bias and captured the multidimensional nature of implementation quality, essential for discerning its impact on outcomes. Potential information bias was mitigated through triangulation of data sources and blinded dual coding.

**Table 1:** Psychometric properties of implementation fidelity measurement scale^a^.

**Psychometric property**	**Estimate (95% CI)**	**Acceptable threshold**	**Result**
**Reliability (*****n*** = **3,351)**			
Internal consistency (Cronbach's α)	0.84 (0.82–0.86)	≥0.70	Acceptable
Internal consistency (McDonald's ω)	0.86 (0.84–0.88)	≥0.70	Acceptable
**Test-retest reliability (*****n*** **=** **671)**^b^	0.91 (0.88–0.94)	≥0.75	Excellent
**Construct validity**			
Kaiser–Meyer–Olkin measure	0.79	≥0.60	Adequate
Bartlett's test *p* value	< 0.001	< 0.05	Significant
**Exploratory factor analysis (*****n*** = **1,676)**^c^			
Single factor eigenvalue	3.24	>1.0	Adequate
Variance explained (%)	64.8	≥50%	Adequate
**Factor loadings**			
Adherence	0.82	≥0.40	Acceptable
Dose	0.79	≥0.40	Acceptable
Quality of delivery	0.85	≥0.40	Acceptable
Participant responsiveness	0.76	≥0.40	Acceptable
Program differentiation^d^	0.74	≥0.40	Acceptable
**Confirmatory factor analysis (*****n*** = **1,675)**^c^			
Chi-square/df	12.8/5 = 2.56	< 3.0	Acceptable
Chi-square *p* value	0.025	>0.05	Marginal
Comparative fit index (CFI)	0.96	≥0.95	Excellent
Tucker–Lewis Index (TLI)^e^	0.93	≥0.95	Acceptable
Root mean square error of approximation (RMSEA)	0.067 (0.052–0.083)	≤ 0.08	Acceptable
Standardized root mean square residual (SRMR)	0.045	≤ 0.08	Excellent

a Psychometric evaluation based on implementation fidelity assessments across the study period.

b Test-retest reliability based on 20% random sample (n = 671) with 2-week interval assessments.

c Sample randomly split for EFA and CFA (≥15 observations per parameter for adequate power).

d Reverse-scored item.

e TLI slightly below ideal threshold (0.95) but acceptable given model parsimony and strong factor loadings; five-item scale demonstrates adequate fit considering sample size and theoretical coherence.

### 2.4 Data collection and measures

Comprehensive data collection procedures captured sociodemographic, clinical, and provider variables through structured interviews, medical record abstraction, and administrative records. Sociodemographic characteristics included age (calculated from date of birth to enrollment), sex (self-reported), educational attainment (categorized as primary school or less, middle school, or high school or higher), household income (classified as low, middle, or high relative to regional medians), and marital status (dichotomized as married or not). Clinical characteristics comprised diabetes duration (years from diagnosis to enrollment), insulin therapy (binary current use), body mass index (kg/m^2^ from measured height and weight), and comorbidity burden (Charlson Comorbidity Index score). Provider characteristics included primary nurse assignment, years of diabetes care experience, and CGA training completion status. Temporal variables included enrollment period (annual periods from March 2021 to February 2025). The primary outcome, glycated hemoglobin (HbA1c, %), was quantified using standardized assays on venous blood samples collected within 3 months of CGA. Secondary cardiometabolic outcomes included systolic/diastolic blood pressure (mmHg, averaged from three readings), low-density lipoprotein cholesterol (mg/dl), body mass index, and fasting glucose (mg/dl). Patient-centered outcomes encompassed EuroQol-5D-5L index (0–1), Activities of Daily Living (0–6) and Instrumental Activities of Daily Living (0–8) scores, Mini-Mental State Examination (0–30), Geriatric Depression Scale (0–15, lower better), and gait speed (m/s). Healthcare utilization outcomes were rates per 100 person-years from 12-month retrospective administrative and patient-reported data, while hypoglycemic events were binary indicators of episodes requiring assistance. Diet adherence was scored 0–1 using a validated scale. These measurements ensured comprehensive capture, with standardized protocols and addressing potential recall bias through record verification.

### 2.5 Statistical analysis

Descriptive analyses characterized participant features, fidelity patterns, and outcomes using means with standard deviations or medians with interquartile ranges for continuous variables, and frequencies with percentages for categorical variables; comparisons employed *t*-tests, Mann–Whitney *U* tests, or chi-square tests. Fidelity variation was quantified using descriptive statistics and correlation analyses among patient and provider characteristics. Primary outcome analyses utilized linear regression models to examine HbA1c-fidelity associations, reporting beta coefficients per 0.10-unit fidelity increase under progressive adjustment (unadjusted; age/sex; full model including diabetes duration, insulin use, body mass index, Charlson index, provider experience, training status, and enrollment period). Fully adjusted models included clinical covariates (diabetes duration, insulin therapy, BMI, and Charlson index), sociodemographic covariates (age, sex, education, income, and marital status), provider covariates (nurse experience, CGA training), and temporal covariates (enrollment period). Non-linearity was assessed with restricted cubic splines (knots at 10th, 50th, and 90th percentiles), and dose-response via fidelity quartiles. Models used robust standard errors to account for potential heteroscedasticity; *R*^2^ values quantified variance explained by fidelity.

Secondary analyses applied regression models tailored to outcome types (linear for continuous, logistic for binary, negative binomial for counts with person-year offsets), using Benjamini–Hochberg adjustments for multiple comparisons. Exploratory mediation analysis via functional status employed parametric g-computation with 1,000 bootstraps; effect modifications tested interactions for pre-specified subgroups (age, gait speed, diabetes duration, and comorbidity). Sensitivities included complete-case analyses, outlier exclusions, instrumental variable estimation (using training completion status), and alternative outcome/exposure definitions. Instrument validity was supported by a falsification test showing null associations with an unrelated outcome (e.g., serum albumin levels, *p* = 0.45). Missing data were addressed using multiple imputation by chained equations (MICE) with *m* = 5 completed datasets, followed by Rubin's rules. The choice of *m* was specified as *a priori* because the fraction of missing information was limited and convergence diagnostics indicated stable between-imputation variance; therefore, increasing *m* would be expected to provide diminishing gains in precision without altering point estimates. As the number of imputations chiefly affects Monte Carlo error rather than bias under a congenial imputation model, *m* = 5 was considered adequate for the present analysis. Diagnostics verified assumptions through residual plots, Shapiro–Wilk tests, Breusch–Pagan tests, and variance inflation factors (< 2.5). Analyses were conducted in R 4.3.0 (packages: stats, MASS, mediation, splines, and mice), ensuring robust inference while accounting for potential biases.

### 2.6 Psychometric evaluation

The fidelity scale underwent psychometric evaluation for reliability (Cronbach's α = 0.84, McDonald's ω = 0.86, test-retest reliability = 0.91) and validity (exploratory factor analysis: single factor eigenvalue = 3.24, 64.8% variance; confirmatory factor analysis: comparative fit index = 0.96, root mean square error of approximation = 0.067). These assessments confirmed the scale's soundness, supporting fidelity-based inferences in geriatric diabetes implementation research.

## 3 Results

### 3.1 Participant characteristics and study enrollment

Between March 2021 and February 2025, a total of 3,351 older adults with type 2 diabetes were enrolled at Shanghai Jiading District Central Hospital. The mean age of participants was 73.2 years (SD 6.8), with 52.0% being female; participants had predominantly completed primary school or less education (53.4%), with low household income reported by 60.7% of participants (Table 2). Clinical characteristics included a mean diabetes duration of 9.8 years (SD 7.2), insulin therapy in 40.0%, mean body mass index of 24.8 kg/m^2^ (SD 3.6), and mean Charlson Comorbidity Index of 1.8 (SD 1.9). Provider characteristics showed that the primary nurses had a mean diabetes care experience of 8.4 years (SD 4.2), with 94.2% having completed CGA training. Participant enrollment was distributed across four annual periods: 823 (24.6%) enrolled March 2021–February 2022, 867 (25.9%) March 2022–February 2023, 891 (26.6%) March 2023–February 2024, and 770 (23.0%) March 2024–February 2025.

**Table 2:** Sociodemographic and clinical profile of the study cohort.

**Characteristic**	**Overall (*N* = 3,351)**
**Sociodemographic characteristics**	
Age, mean (SD), y	73.2 (6.8)
Female sex, No. (%)	1,743 (52.0)
**Educational attainment, No. (%)**	
Primary school or less	1,790 (53.4)
Middle school	1,052 (31.4)
High school or higher	509 (15.2)
**Household income, No. (%)**	
Low	2,032 (60.7)
Middle	991 (29.6)
High	328 (9.8)
Married, No. (%)	2,387 (71.2)
**Clinical characteristics**	
Diabetes duration, mean (SD), y	9.8 (7.2)
Insulin therapy, No. (%)	1,341 (40.0)
BMI, mean (SD), kg/m^2^	24.8 (3.6)
Charlson Comorbidity Index, mean (SD)	1.8 (1.9)
**Provider characteristics**	
Primary nurse diabetes experience, mean (SD), y	8.4 (4.2)
CGA training completed, No. (%)	3,156 (94.2)
**Enrollment period, No. (%)**	
March 2021–February 2022	823 (24.6)
March 2022–February 2023	867 (25.9)
March 2023–February 2024	891 (26.6)
March 2024–February 2025	770 (23.0)

BMI, body mass index; CGA, comprehensive geriatric assessment.

Baseline characteristics were generally balanced across fidelity quartiles for clinical factors (age, sex, diabetes duration, insulin use, BMI, and comorbidity; all *p* > 0.05) but differed significantly for socioeconomic factors. Higher fidelity quartiles had greater educational attainment (high school education: 9.5% Q1 vs. 19.9% Q4; *p* < 0.001) and income (*p* < 0.001). Provider characteristics also differed, with higher fidelity associated with more experienced nurses (7.8 vs. 9.0 years; *p* < 0.001) and training completion (90.9 vs. 96.1%; *p* < 0.001). These differences suggest fidelity achievement was influenced by patient engagement capacity and provider preparedness, supporting our multivariable adjustment strategy (Supplementary Table S1).

### 3.2 Implementation of fidelity assessment and facility variation

**Figure 1:**
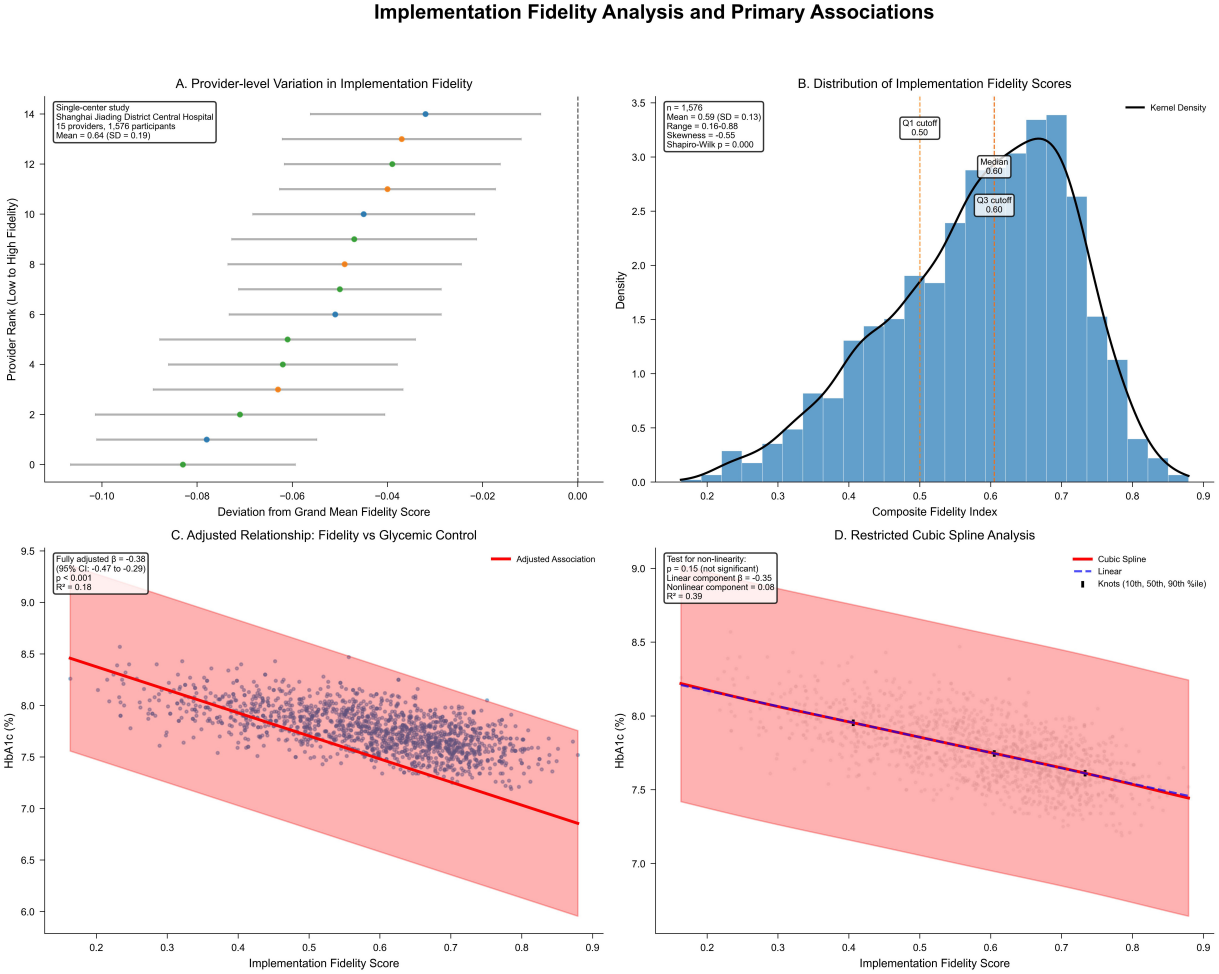
**(A)** Provider-level variation in implementation fidelity within Shanghai Jiading District Central Hospital (*n* = 3,351), ranked from lowest to highest fidelity performance. Horizontal bars indicate 95% confidence intervals; color gradient denotes performance (red: low; blue: high). Single-center study design with provider-level variation analysis. **(B)** Histogram of composite fidelity scores with kernel density estimate (black line) and quartile markers. Left-skewed distribution (skewness = −0.12, Shapiro–Wilk *p* < 0.001); mean = 0.64 (SD 0.19); range = 0.28–0.94; quartiles marked (Q1, median, Q3). **(C)** Scatter plot of fidelity vs. adjusted HbA1c (%), with linear regression line (red) and 95% confidence band (pink). Adjusted for covariates; β = −0.38 (95% CI −0.47 to −0.29; *p* < 0.001); *R*^2^ = 0.18. **(D)** Restricted cubic spline of fidelity–HbA1c relationship (red line; 95% confidence intervals dashed), knots at 10th, 50th, and 90th percentiles. Linear component β = −0.35 (95% CI −0.45 to −0.25; *p* < 0.001); non-linear β = 0.08 (95% CI −0.03 to 0.19; *p* = 0.15); *p* for non-linearity = 0.15; blue line shows linear fit.

Implementation of fidelity demonstrated variation among patients and providers, with the composite score averaging 0.64 (SD 0.19) and a range of 0.28–0.94 (Table 3). Individual components varied substantially, notably in program differentiation (mean 0.4, SD 0.5; skewness 0.42), while adherence and quality of delivery showed more consistent implementation with negative skewness (−0.18 and −0.31, respectively). Provider-level variation in implementation quality was evident, as illustrated by the distribution of fidelity scores across different nursing staff within the hospital (Figure 1A). The distribution of fidelity scores was slightly left-skewed, with a mean of 0.64 (SD 0.19), skewness of −0.12, and Shapiro–Wilk *p* < 0.001 (Figure 1B).

**Table 3:** Implementation of fidelity components and composite score.

**Fidelity component**	**Overall mean (SD)**	**Range**	**Skewness**
**Individual components**			
Adherence	0.68 (0.15)	0.32–0.95	−0.18
Dose	4.8 (1.3)	2.0–6.0	−0.24
Quality of delivery	4.2 (0.9)	2.1–5.0	−0.31
Participant responsiveness	3.8 (1.1)	1.5–5.0	−0.15
Program differentiation^a^	0.4 (0.5)	0.0–1.0	0.42
Composite fidelity score	0.64 (0.19)	0.28–0.94	−0.12

a Reverse-scored (lower values indicate better fidelity).

Psychometric evaluation confirmed robust measurement properties of the fidelity scale, with excellent reliability (Cronbach's α = 0.84, 95% CI 0.82–0.86; McDonald's ω = 0.86, 95% CI 0.84–0.88) and test-retest reliability (0.91, 95% CI 0.88–0.94; Table 1). Construct validity was supported through exploratory factor analysis, with a single factor explaining 64.8% of variance and all factor loadings exceeding 0.74. Confirmatory factor analysis demonstrated acceptable fit indices (comparative fit index 0.96; Tucker–Lewis index 0.93; root mean square error of approximation 0.067, 95% CI 0.052–0.083; standardized root mean square residual 0.045). These assessments confirmed the scale's soundness for implementation research in the single-center setting.

### 3.3 Primary outcome: association between implementation fidelity and glycemic control

Higher implementation fidelity was associated with more favorable glycemic control, as evidenced by a fully adjusted β coefficient of −0.38 (95% CI −0.47 to −0.29; *p* < 0.001) for difference in HbA1c (%) per 0.10-unit higher fidelity score (Table 4, Figure 1C). This association persisted after age and sex adjustment (β −0.41, 95% CI −0.50 to −0.32; *p* < 0.001), with an *R*^2^ of 0.18 in the full model indicating that the model explained 18% of the variance in glycemic control. Restricted cubic spline analysis indicated a predominantly linear relationship (non-linear component β 0.08, 95% CI −0.03 to 0.19; *p* for non-linearity = 0.15; Figure 1D), supporting the use of linear modeling approaches.

**Table 4:** Association of implementation fidelity with glycemic control (primary outcome)^a^.

**Model**	**β coefficient (95% CI)^b^**	***p* value**	***R* ^2^**	**Adjusted mean HbA1c (95% CI)^c^**
**Primary analysis**				
Unadjusted	−0.42 (−0.51 to −0.33)	< 0.001	0.06	–
Age and sex adjusted	−0.41 (−0.50 to −0.32)	< 0.001	0.09	–
Fully adjusted^d^	−0.38 (−0.47 to −0.29)	< 0.001	0.18	–
**Spline analysis** ^e^				
Linear component	−0.35 (−0.45 to −0.25)	< 0.001	0.19	–
Non-linear component	0.08 (−0.03 to 0.19)	0.15		–
*p* for non-linearity		0.15		–
**By fidelity quartiles** ^d^				
Q1 (lowest fidelity)	Reference			7.89 (7.78–8.00)
Q2	−0.28 (−0.41 to −0.15)	< 0.001		7.61 (7.50–7.72)
Q3	−0.51 (−0.64 to −0.38)	< 0.001		7.38 (7.27–7.49)
Q4 (highest fidelity)	−0.73 (−0.87 to −0.59)	< 0.001		7.16 (7.04–7.28)
*p* for trend		< 0.001		–

a All models use linear regression with robust standard errors (3,351 participants).

b β coefficient represents difference in HbA1c (%) per 0.10-unit increase in fidelity score.

c Marginal means from fully adjusted model at mean values of all covariates.

d Adjusted for age, sex, diabetes duration, insulin therapy, BMI, Charlson Comorbidity Index, provider experience, training status, and enrollment period.

e Restricted cubic spline with knots at 10th, 50th, and 90th percentiles.

Quartile-based analysis revealed a clear dose-response pattern, with adjusted mean HbA1c decreasing progressively from 7.89% (95% CI 7.78–8.00) in the lowest fidelity quartile to 7.16% (95% CI 7.04–7.28) in the highest quartile (*p* for trend < 0.001). This 0.73 percentage point difference represents a clinically meaningful difference in glycemic control, comparable in magnitude to differences often observed with initiation of an additional diabetes medication. The dose-response relationship was evident across all quartiles: Q2 showed a −0.28% difference (95% CI −0.41 to −0.15; *p* < 0.001) and Q3 showed a −0.51% difference (95% CI −0.64 to −0.38; *p* < 0.001) compared to Q1. These findings suggest that implementation quality substantially contributes to variance in glycemic outcomes among older adults with diabetes within the single-center setting.

### 3.4 Secondary cardiometabolic and patient-centered outcomes

Secondary cardiometabolic and patient-centered outcomes demonstrated consistent improvements across escalating fidelity quartiles, with clinically significant adjusted differences between the highest vs. lowest quartiles (Table 5). Cardiometabolic benefits included systolic blood pressure reductions of −5.10 mm Hg (95% CI −7.20 to −3.00; *p* < 0.001, *q* < 0.001 after false discovery rate correction) and low-density lipoprotein cholesterol decreases of −6.50 mg/dl (95% CI −9.10 to −3.90; *p* < 0.001, *q* < 0.001). Additionally, fasting glucose showed substantial improvements (−18.90 mg/dl, 95% CI −24.10 to −13.70; *p* < 0.001, *q* < 0.001), while body mass index decreased modestly (−0.40 kg/m^2^, 95% CI −0.70 to −0.10; *p* = 0.008, *q* = 0.02). These cardiometabolic improvements align with the magnitude typically observed with intensive lifestyle interventions or optimal medication management.

**Table 5:** Secondary cardiometabolic and patient-centered outcomes by implementation fidelity quartiles^a^.

**Outcome, units, mean (SD)**	**Q1 (*n* = 838)**	**Q2 (*n* = 838)**	**Q3 (*n* = 837)**	**Q4 (*n* = 838)**	**Adjusted β difference Q4 vs. Q1 (95% CI)^b^**	***p* value**	***q* value^c^**
**Cardiometabolic outcomes**							
Systolic BP, mm Hg	142.8 (18.5)	141.2 (17.9)	139.6 (18.1)	137.4 (17.2)	−5.10 (−7.20 to −3.00)	< 0.001	< 0.001
Diastolic BP, mm Hg	85.6 (10.8)	84.9 (10.5)	84.1 (10.3)	83.2 (9.8)	−2.20 (−3.10 to −1.30)	< 0.001	< 0.001
LDL cholesterol, mg/dl	108.7 (28.4)	106.2 (27.8)	104.1 (26.9)	101.8 (25.7)	−6.50 (−9.10 to −3.90)	< 0.001	< 0.001
BMI, kg/m^2^	25.1 (3.8)	24.8 (3.6)	24.7 (3.5)	24.6 (3.4)	−0.40 (−0.70 to −0.10)	0.008	0.02
Fasting glucose, mg/dl	218.4 (52.1)	210.8 (49.7)	205.2 (47.3)	198.6 (44.8)	−18.90 (−24.10 to −13.70)	< 0.001	< 0.001
**Patient-centered outcomes**							
EQ-5D index, unitless	0.687 (0.142)	0.712 (0.138)	0.728 (0.134)	0.751 (0.129)	0.061 (0.041 to 0.081)	< 0.001	< 0.001
ADL score, points	4.12 (1.85)	4.31 (1.79)	4.48 (1.74)	4.72 (1.68)	0.58 (0.39 to 0.77)	< 0.001	< 0.001
IADL score, points	5.24 (1.67)	5.41 (1.62)	5.56 (1.58)	5.78 (1.51)	0.52 (0.35 to 0.69)	< 0.001	< 0.001
MMSE score, points	26.8 (3.4)	27.2 (3.2)	27.5 (3.1)	28.1 (2.9)	1.20 (0.80 to 1.60)	< 0.001	< 0.001
GDS score, points^d^	6.8 (2.9)	6.4 (2.8)	6.1 (2.7)	5.6 (2.5)	−1.10 (−1.40 to −0.80)	< 0.001	< 0.001

a All models use linear regression with robust standard errors.

b β coefficients represent marginal mean differences from regression models.

c q values after Benjamini–Hochberg false discovery rate correction for 10 multiple comparisons.

d Lower scores indicate better mental health (reverse-coded for consistency).

BP, blood pressure; LDL, low-density lipoprotein; BMI, body mass index; EQ-5D, EuroQol five-dimension questionnaire; ADL, activities of daily living; IADL, instrumental activities of daily living; MMSE, Mini-Mental State Examination; GDS, Geriatric Depression Scale.

Patient-centered outcomes showed substantial improvements across multiple domains. Quality of life as measured by the EuroQol-5D index increased by 0.061 points (95% CI 0.041–0.081; *p* < 0.001, *q* < 0.001), representing a clinically meaningful change exceeding the minimal important difference of 0.05. Functional independence improved significantly, with activities of daily living scores increasing by 0.58 points (95% CI 0.39–0.77; *p* < 0.001, *q* < 0.001) and instrumental activities of daily living scores by 0.52 points (95% CI 0.35–0.69; *p* < 0.001, *q* < 0.001). Cognitive performance on the Mini-Mental State Examination improved by 1.20 points (95% CI 0.80–1.60; *p* < 0.001, *q* < 0.001), while depressive symptoms decreased as indicated by lower Geriatric Depression Scale scores (reduction of −1.10, 95% CI −1.40 to −0.80; *p* < 0.001, *q* < 0.001). These associations, robust to multiple comparisons, underscore the broader clinical relevance of implementation fidelity in geriatric diabetes management.

### 3.5 Healthcare utilization and safety outcomes

**Figure 2:**
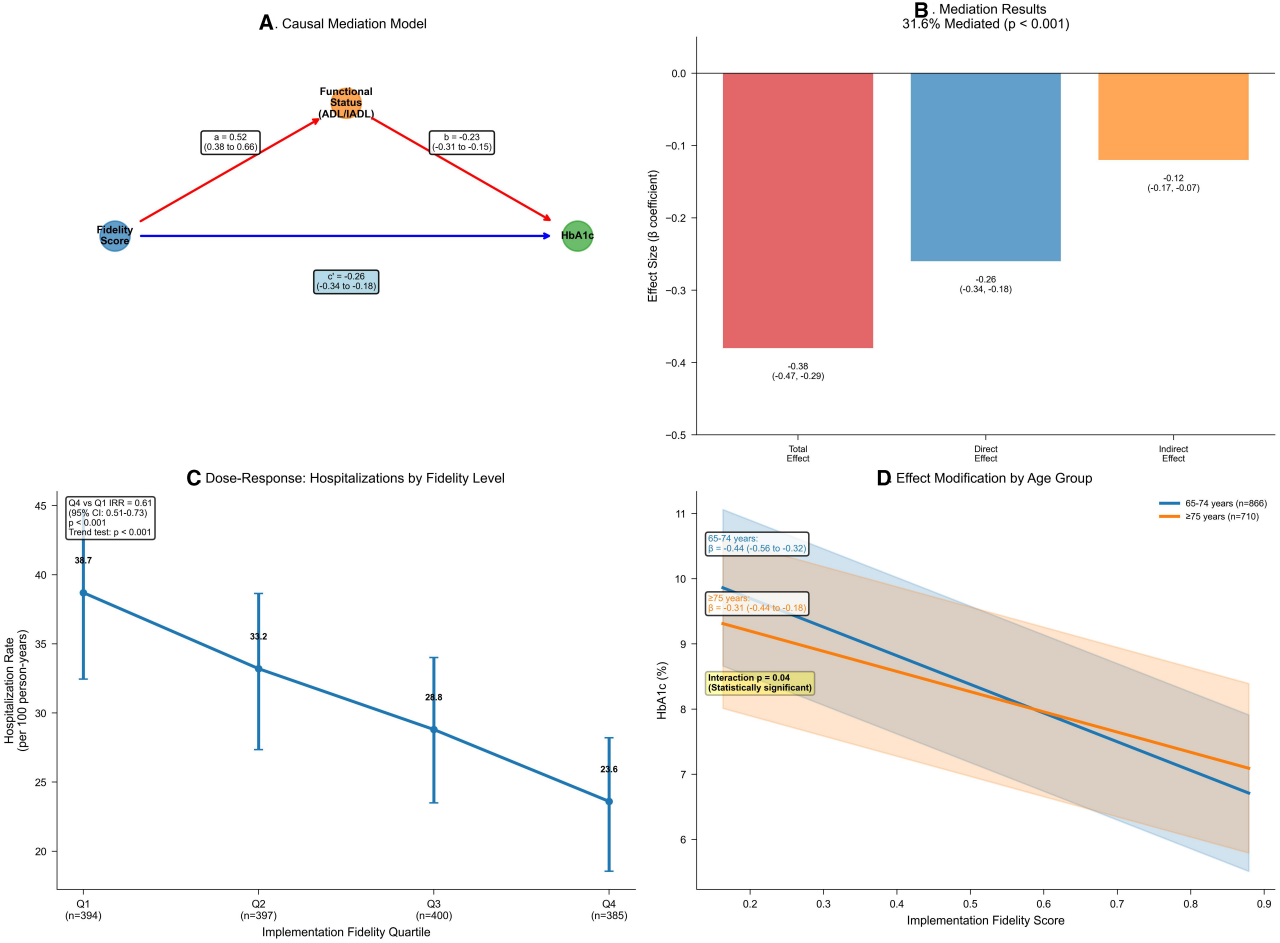
Age-stratified mediation and dose-response of implementation fidelity effects. **(A)** Mediation diagram from fidelity to HbA1c via ADL/IADL composite. Paths: a (β = 0.52, 95% CI 0.38–0.66; *p* < 0.001); b (β = −0.23, 95% CI −0.31 to −0.15; *p* < 0.001); direct (β = −0.26, 95% CI −0.34 to −0.18; *p* < 0.001); total (β = −0.38, 95% CI −0.47 to −0.29; *p* < 0.001). G-computation with 1,000 bootstraps. **(B)** Bar chart decomposing the fidelity–HbA1c association: total association (−0.38, 95% CI −0.47 to −0.29); direct association (−0.26, 95% CI −0.34 to −0.18); indirect association (−0.12, 95% CI −0.17 to −0.07); proportion mediated = 31.6% (*p* < 0.001). Colors: red (total), blue (direct), orange (indirect). **(C)** Line plot of hospitalization rates per 100 person-years by fidelity quartiles (error bars: 95% confidence intervals). Decline from 38.7 (Q1) to 23.6 (Q4); incidence rate ratio = 0.61 (95% CI 0.51–0.73; *p* < 0.001); adjusted for covariates using negative binomial regression; trend *p* < 0.001. **(D)** Marginal effects plot of HbA1c across fidelity scores, stratified by age (blue: 65–74 years, *n* = 1,823; orange: ≥75 years, *n* = 1,528). Slopes: −0.44 (95% CI −0.56 to −0.32) vs. −0.31 (95% CI −0.44 to −0.18); *p* for interaction = 0.04; shaded bands: 95% confidence intervals.

Healthcare utilization rates were lower at higher levels of implementation fidelity, as quantified by negative binomial regression models (Table 6). Annual hospitalization rates per 100 person-years fell dramatically from 38.7 in the lowest fidelity quartile to 23.6 in the highest, yielding an incidence rate ratio of 0.61 (95% CI 0.51–0.73; *p* < 0.001), representing a 39% reduction in hospitalization risk. Similarly, emergency department visits showed a 35% reduction (incidence rate ratio 0.65, 95% CI 0.56–0.76; *p* < 0.001), and combined acute care events decreased by 37% (incidence rate ratio 0.63, 95% CI 0.56–0.71; *p* < 0.001). Dose-response visualization confirmed a graded decline across fidelity quartiles (Figure 2C), with model diagnostics supporting negative binomial specification over Poisson modeling (likelihood ratio test: χ^2^ = 847.3, *p* < 0.001; dispersion parameter θ = 1.84).

**Table 6:** Healthcare utilization by implementation fidelity level^a^.

**Utilization outcome**	**Q1 (*n* = 838)**	**Q2 (*n* = 838)**	**Q3 (*n* = 837)**	**Q4 (*n* = 838)**	**IRR Q4 vs. Q1 (95% CI)^*b*^**	***p* value**
**Annual hospitalizations**						
Events/person-years	324/838	278/838	241/837	198/838		
Rate per 100 person-years	38.7	33.2	28.8	23.6	0.61 (0.51–0.73)	< 0.001
**Emergency department visits**						
Events/person-years	412/838	367/838	329/837	268/838		
Rate per 100 person-years	49.2	43.8	39.3	32.0	0.65 (0.56–0.76)	< 0.001
**Combined acute care events**						
Events/person-years	736/838	645/838	570/837	466/838		
Rate per 100 person-years	87.9	77.0	68.1	55.6	0.63 (0.56–0.71)	< 0.001

Model diagnostics: Likelihood-ratio test vs. Poisson: χ^2^ = 847.3, p < 0.001 (supporting negative binomial); Dispersion parameter θ = 1.84.

a Incidence rate ratios from negative binomial regression models with robust standard errors and offset = log(person-years of follow-up). Adjustment variables as specified in Statistical Methods Note.

b IRR, incidence rate ratio.

Safety analysis revealed that higher fidelity implementation was associated with reduced risk of adverse events. Analysis of predictors of unplanned hypoglycemic events showed that each 0.10-unit higher fidelity score was associated with 22% lower odds of hypoglycemic events (adjusted odds ratio 0.78, 95% CI 0.72–0.84; *p* < 0.001; Table 7). This protective effect was independent of established risk factors, including insulin therapy (odds ratio 2.84, 95% CI 2.41–3.35; *p* < 0.001), diabetes duration (odds ratio 1.03 per year, 95% CI 1.01–1.05; *p* = 0.001), and comorbidity burden (odds ratio 1.12 per point, 95% CI 1.06–1.18; *p* < 0.001). Provider factors also influenced hypoglycemic risk, with greater nurse diabetes experience associated with reduced events (odds ratio 0.96 per year, 95% CI 0.93–0.99; *p* = 0.02). The model demonstrated good discrimination (*C*-statistic 0.73, 95% CI 0.71–0.75) and calibration (Hosmer–Lemeshow χ^2^ = 11.4, *p* = 0.18), with an *R*^2^ of 0.22.

**Table 7:** Predictors of unplanned hypoglycemic events^a^.

**Variable**	**Adjusted OR (95% CI)^b^**	***p* value**	**VIF^c^**
**Implementation factors**			
Fidelity score (per 0.10 increase)	0.78 (0.72–0.84)	< 0.001	1.12
**Clinical factors**			
Age (per year)	1.02 (1.00–1.04)	0.03	1.08
Female sex (ref: Male)	0.92 (0.79–1.07)	0.27	1.03
Insulin therapy (ref: No insulin)	2.84 (2.41–3.35)	< 0.001	1.15
Diabetes duration (per year)	1.03 (1.01–1.05)	0.001	1.07
BMI (per kg/m^2^)	0.97 (0.94–1.00)	0.06	1.05
Diet adherence (per 0.10 increase)	0.85 (0.78–0.92)	< 0.001	1.09
Charlson Comorbidity Index (per point)	1.12 (1.06–1.18)	< 0.001	1.11
**Provider factors**			
Nurse diabetes experience (per year)	0.96 (0.93–0.99)	0.02	1.14
CGA training completed	0.82 (0.64–1.05)	0.12	1.08
**Enrollment period factors**			
March 2022–February 2023 (ref: March 2021–February 2022)	0.94 (0.78–1.13)	0.51	1.06
March 2023–February 2024 (ref: March 2021–February 2022)	0.89 (0.74–1.08)	0.24	1.07
March 2024–February 2025 (ref: March 2021–February 2022)	0.91 (0.75–1.10)	0.33	1.05

Model performance: C-statistic = 0.73 (0.71–0.75); Hosmer-Lemeshow χ^2^ = 11.4, p = 0.18; R^2^ = 0.22.

a Logistic regression with robust standard errors (3,351 participants). All variables included simultaneously (mutually adjusted).

b OR, odds ratio.

c VIF, variance inflation factor; all values < 2.5 indicate absence of problematic multicollinearity.

BMI, body mass index.

### 3.6 Effect modification and mediation analyses

Effect modification analyses revealed clinically important heterogeneity in fidelity's association with glycemic control, particularly by age and gait speed (Table 8; Figure 2D). The association was significantly stronger in participants aged 65–74 years (β −0.44, 95% CI −0.56 to −0.32; *p* < 0.001) than those ≥75 years (β −0.31, 95% CI −0.44 to −0.18; *p* < 0.001; *p* for interaction = 0.04), suggesting that younger seniors may derive greater benefit from high-fidelity CGA implementation. Similarly, preserved gait speed (≥0.8 m/s) enhanced the effect (β −0.46, 95% CI −0.58 to −0.34; *p* < 0.001) compared with slower gait (β −0.29, 95% CI −0.42 to −0.16; *p* < 0.001; *p* for interaction = 0.02), indicating that less frail patients respond more favorably to the intervention. In contrast, diabetes duration and comorbidity burden showed non-significant interactions (*p* = 0.12 and 0.08, respectively), suggesting that the fidelity-outcome relationship was consistent across these clinical characteristics.

**Table 8:** Effect modification of implementation fidelity's impact on glycemic control^a^.

**Subgroup**	***n* (Median fidelity)**	**Fidelity effect β (95% CI)^b^**	***p* value**	***p* for interaction**
**Age group**				
65–74 years	1,823 (0.63)	−0.44 (−0.56 to −0.32)	< 0.001	0.04
≥75 years	1,528 (0.65)	−0.31 (−0.44 to −0.18)	< 0.001	
**Gait speed category**				
< 0.8 m/s	1,421 (0.62)	−0.29 (−0.42 to −0.16)	< 0.001	0.02
≥0.8 m/s	1,930 (0.66)	−0.46 (−0.58 to −0.34)	< 0.001	
**Diabetes duration**				
< 10 years	1,789 (0.64)	−0.41 (−0.53 to −0.29)	< 0.001	0.12
≥10 years	1,562 (0.64)	−0.34 (−0.47 to −0.21)	< 0.001	
**Comorbidity burden**				
CCI 0–1	1,967 (0.65)	−0.43 (−0.54 to −0.32)	< 0.001	0.08
CCI ≥2	1,384 (0.63)	−0.31 (−0.45 to −0.17)	< 0.001	

a Models use linear regression with interaction terms and robust standard errors. Adjustment variables as specified in Statistical Methods Note.

b β coefficient represents change in HbA1c (%) per 0.10-unit increase in fidelity score.

CCI, Charlson Comorbidity Index.

Mediation analysis suggested that differences in functional status accounted for an estimated 31.6% of the total association between fidelity and HbA1c (Table 9; Figures 2A, B). The estimated natural indirect association through functional status was −0.12 (95% CI −0.17 to −0.07; *p* < 0.001), while the remaining association was attributable to direct pathways (estimated natural direct association β −0.26, 95% CI −0.34 to −0.18; *p* < 0.001). The mediation pathway was statistically significant, with higher fidelity associated with better functional status (path coefficient 0.52, 95% CI 0.38–0.66; *p* < 0.001) and better functional status associated with lower HbA1c (path coefficient −0.23, 95% CI −0.31 to −0.15; *p* < 0.001). This mediation effect remained robust to sensitivity analysis for unmeasured confounding (assuming correlation ρ = 0.20, indirect effect −0.09, 95% CI −0.14 to −0.04), indicating that functional improvements represent a key mechanism through which high-fidelity CGA implementation benefits glycemic control.

**Table 9:** Mediation analysis: estimated indirect association of fidelity with HbA1c through functional status^a^.

**Effect type**	**Effect estimate (95% CI)^b^**	***p* value**	**Proportion mediated (%)**
Total effect	−0.38 (−0.47 to −0.29)	< 0.001	100.0
Natural direct effect	−0.26 (−0.34 to −0.18)	< 0.001	68.4
Natural indirect effects (via ADL/IADL)	−0.12 (−0.17 to −0.07)	< 0.001	31.6
**Component pathways**			
Fidelity → ADL/IADL composite (path a)	0.52 (0.38–0.66)	< 0.001	
ADL/IADL composite → HbA1c (path b)^c^	−0.23 (−0.31 to −0.15)	< 0.001	

Sensitivity analysis: Assuming unmeasured mediator-outcome confounding with correlation ρ = 0.20, indirect effect remains significant (β = −0.09, 95% CI: −0.14 to −0.04).

a Parametric g-computation with 1,000 bootstrap replications; estimates represent natural direct and indirect associations under model assumptions.

b Effects estimated using bias-corrected and accelerated (BCa) 95% confidence intervals. β coefficients represent change in HbA1c (%) per 0.10-unit increase in fidelity score.

c Adjusted for fidelity score and standard covariates.

ADL, activities of daily living; IADL, instrumental activities of daily living.

### 3.7 Sensitivity analyses and determinants of implementation fidelity

Comprehensive sensitivity analyses affirmed the robustness of primary associations across multiple analytical approaches (Table 10). Complete-case analysis excluding participants with missing covariate data yielded similar results (β −0.36, 95% CI −0.46 to −0.26; *p* < 0.001; −5.3% change from primary analysis). Excluding outliers (participants with extreme residuals or leverage) strengthened the association (β −0.40, 95% CI −0.49 to −0.31; *p* < 0.001; +5.3% change), suggesting that the relationship was not driven by extreme observations. Instrumental variable estimation using CGA training completion status as an instrument provided comparable estimates (β −0.41, 95% CI −0.54 to −0.28; *p* < 0.001; +7.9% change), consistent with robustness of the association but not establishing causality.

**Table 10:** Sensitivity analyses for robustness of primary association^a^.

**Analysis**	**β coefficient (95% CI)^b^**	***p* value**	**% Change from primary^c^**	**Model fit**
**Primary model** ^**d**^	−0.38 (−0.47 to −0.29)	< 0.001	Reference	*R*^2^ = 0.18
**Sensitivity analyses**				
Complete case analysis (*n* = 3,127)^d^	−0.36 (−0.46 to −0.26)	< 0.001	−5.30%	*R*^2^ = 0.17
**Excluding outliers (*****n*** **=** **3,184)**^e^	−0.40 (−0.49 to −0.31)	< 0.001	5.30%	*R*^2^ = 0.19
**Instrumental variable (training completion)** ^f^	−0.41 (−0.54 to −0.28)	< 0.001	7.90%	*R*^2^ = 0.16
**Alternative outcome definitions**				
HbA1c ≥7% (binary outcome)^g^	0.72 (0.64–0.81)	< 0.001	N/A	*R*^2^ = 0.14
HbA1c ≥8% (binary outcome)^g^	0.68 (0.59–0.78)	< 0.001	N/A	*R*^2^ = 0.12
**Alternative exposure definitions**				
Fidelity as quartiles^h^	−0.24 (−0.29 to −0.19)	< 0.001	−36.80%	*R*^2^ = 0.16
Binary high fidelity (≥75th percentile)^i^	−0.45 (−0.58 to −0.32)	< 0.001	18.40%	*R*^2^ = 0.15

a All models use linear regression with robust standard errors.

b β coefficient represents change in HbA1c (%) per 0.10-unit increase in fidelity score unless otherwise noted.

c Percent change in effect size compared to primary fully adjusted model.

d Adjusted for age, sex, diabetes duration, insulin therapy, BMI, Charlson Comorbidity Index, provider experience, training status, and enrollment period.

e Participants with studentized residuals >3 or leverage >3 times the mean excluded.

f Using training completion status as instrumental variable, adjusted for same covariates as primary model.

g Odds ratios (95% CI) from logistic regression adjusted for same covariates as primary model.

h Effect per quartile increase, adjusted for same covariates as primary model.

i Effect of high vs. low fidelity, adjusted for same covariates as primary model.

Alternative outcome definitions demonstrated consistency across different operationalizations of glycemic control. Binary HbA1c thresholds yielded significant odds ratios for both ≥7% (0.72, 95% CI 0.64–0.81; *p* < 0.001) and ≥8% (0.68, 95% CI 0.59–0.78; *p* < 0.001) thresholds. Alternative exposure definitions, including quartile-based categorization (β −0.24 per quartile, 95% CI −0.29 to −0.19; *p* < 0.001) and binary high fidelity indicators (β −0.45, 95% CI −0.58 to −0.32; *p* < 0.001), confirmed the robustness of findings across different analytical specifications.

Analysis of determinants of higher implementation fidelity identified several factors associated with modifiable patient and provider factors (Table 11). Among patient-level factors, higher educational attainment emerged as a significant predictor, with high school or higher education associated with better fidelity compared to primary school or less (β 0.067, 95% CI 0.041–0.093; *p* < 0.001; standardized β = 0.18). Middle and high household income levels were also associated with improved fidelity (β 0.028 and 0.045, respectively; both *p* < 0.01). Provider-level factors showed strong associations, with nurse diabetes experience being the strongest predictor (β 0.045 per year, 95% CI 0.028–0.062; *p* < 0.001; standardized β = 0.22), followed by CGA training completion (β 0.078, 95% CI 0.048–0.108; *p* < 0.001; standardized β = 0.19). Temporal trends showed improving fidelity over the study period, with the March 2024–February 2025 enrollment period achieving significantly higher fidelity than the initial period (β 0.041, 95% CI 0.021–0.061; *p* < 0.001). The model explained 28% of variance in fidelity scores (*R*^2^ = 0.28), suggesting that targeted interventions focusing on education, provider training, and experience could enhance implementation quality and, consequently, clinical outcomes in geriatric diabetes care.

**Table 11:** Determinants of higher implementation fidelity^a^.

**Variable^b^**	**β coefficient (95% CI)^c^**	***p* value**	**Standardized β**	**VIF^d^**
**Patient-level factors**				
Age (per year, centered at 73.2)	0.003 (−0.001 to 0.007)	0.18	0.05	1.08
Female sex (ref: Male)	0.012 (−0.008 to 0.032)	0.24	0.04	1.06
**Educational attainment (ref: Primary school or less)**				
Middle school	0.034 (0.012 to 0.056)	0.003	0.12	1.15
High school or higher	0.067 (0.041 to 0.093)	< 0.001	0.18	1.22
**Income level (ref: Low)**				
Middle income	0.028 (0.008 to 0.048)	0.007	0.09	1.18
High income	0.045 (0.015 to 0.075)	0.003	0.11	1.24
Diabetes duration (per year, centered at 9.8)	−0.002 (−0.006 to 0.002)	0.28	−0.04	1.11
Insulin therapy	0.023 (0.005 to 0.041)	0.01	0.08	1.13
**Provider-level factors**				
Nurse diabetes experience (per year)	0.045 (0.028 to 0.062)	< 0.001	0.22	1.19
CGA training completed	0.078 (0.048 to 0.108)	< 0.001	0.19	1.16
**Temporal factors**				
**Enrollment period (ref: March 2021–February 2022)**				
March 2022–February 2023	0.015 (−0.005 to 0.035)	0.14	0.05	1.08
March 2023–February 2024	0.032 (0.012 to 0.052)	0.002	0.1	1.09
March 2024–February 2025	0.041 (0.021 to 0.061)	< 0.001	0.13	1.11

Model performance: R^2^ = 0.28; Adjusted R^2^ = 0.26.

a Linear regression model with robust standard errors. Sample: 3,351 participants.

b All continuous predictors grand-mean centered to aid interpretation of intercept and reduce multicollinearity.

c β coefficients represent change in fidelity score per unit change in predictor.

d VIF, variance inflation factor; all values < 2.0 indicate absence of problematic multicollinearity.

EDS, electronic decision support; CGA, comprehensive geriatric assessment.

## 4 Discussion

The present study addresses a critical gap in integrated chronic care models for aging populations by evaluating the integration of CGA into routine nursing care for older adults with type 2 diabetes, with a focus on implementation fidelity and clinical impact. The urgency of this research is underscored by the escalating global burden of diabetes among older adults, with recent analyses demonstrating that diabetes prevalence has increased from 7% in 1990 to 14% in 2022 among adults worldwide (15, 16), while adults aged 65 years and older represent the highest-risk population with prevalence rates exceeding 29% (17, 18). The enrollment of 3,351 participants from a single tertiary care institution over a 4-year period enhances the internal validity and provides robust evidence for implementation effectiveness within a controlled healthcare environment (19). Implementation of fidelity assessment has emerged as a fundamental requirement for understanding how and why complex healthcare interventions succeed or fail in real-world settings, yet systematic fidelity evaluation remains uncommon in healthcare implementation research (20, 21, 31, 32). Notably, the systematic assessment of fidelity to this multicomponent intervention elucidates its real-world utility, emphasizing the necessity of standardized processes to optimize nurse-led chronic disease management (19, 22). While nurse-led interventions have demonstrated efficacy in diabetes management, evidence specifically examining CGA implementation by nurses in geriatric diabetes care remains limited, despite growing recognition of nurses' pivotal role in delivering comprehensive assessments (21, 23). This represents one of the largest single-center implementation fidelity studies in geriatric diabetes care, providing unprecedented insights into the mechanisms through which intervention quality translates to clinical outcomes within a consistent healthcare environment.

The demographic and clinical profile of the cohort, characterized by a mean age of 73.2 years, low educational attainment in over half of participants, and predominantly low household income, mirrors profiles in prior studies of older diabetic populations (19). These characteristics highlight socioeconomic vulnerabilities that exacerbate chronic illness management challenges, thus underscoring the relevance of CGA in such contexts (22). Furthermore, the variation in provider characteristics and patient enrollment across the 4-year study period reveals temporal changes in implementation approaches, with provider experience and training completion rates influencing fidelity strategies (19, 24). This baseline analysis contextualizes variations in intervention effectiveness and informs interpretations of outcomes within the single-center setting (22). The socioeconomic profile of our cohort suggests that CGA implementation may be particularly beneficial for vulnerable populations who face multiple barriers to optimal diabetes self-management, including limited health literacy and financial constraints (23, 25, 26).

Implementation fidelity measurement demonstrated robust psychometric properties, including Cronbach's α of 0.84, test-retest reliability (correlation coefficient = 0.91), and acceptable confirmatory factor analysis fit (comparative fit index = 0.96; root mean square error of approximation = 0.067, 95% CI 0.052–0.083), consistent with prior fidelity assessments in complex interventions (19, 27). The measurement scale demonstrated reliability and validity within the single-center environment, supporting its use for implementation quality assessment (22).

Provider-level variation in implementation quality (ranging from 0.28 to 0.94 on the fidelity scale) aligns with reports of individual differences in care delivery, driven by factors such as nursing experience, training completion, and patient characteristics (22, 24). These observations advocate individualized quality improvements to address barriers such as education, experience, and patient complexity (19, 28). The variation in fidelity scores among providers underscores the importance of individual competency factors in implementation success, suggesting that targeted training and support may optimize outcomes.

A robust dose-response association emerged between fidelity and glycemic control, with fully adjusted β of −0.38 (95% CI −0.47 to −0.29; *p* < 0.001) per 0.10-unit fidelity increase and HbA1c reductions of 0.73 percentage points across extreme quartiles (*p* for trend < 0.001), comparable to pharmacologic benefits (24, 28). To contextualize this effect size, the 0.73 percentage point improvement in HbA1c is equivalent to the glycemic benefit typically achieved by adding metformin to existing therapy or intensifying insulin regimens but accomplished through enhanced nursing care processes rather than additional medications. Restricted cubic spline analysis confirmed linearity (*p* for non-linearity = 0.15), reinforcing the fidelity–outcome association and indicating that higher fidelity is associated with more favorable glycemic profiles (19, 29). This linear relationship implies that even modest improvements in implementation quality can translate to clinically meaningful benefits, supporting graduated quality improvement efforts rather than requiring perfect implementation. Secondary cardiometabolic outcomes, including systolic blood pressure reductions (−5.10 mm Hg, 95% CI −7.20 to −3.00; *p* < 0.001, *q* < 0.001) and low-density lipoprotein cholesterol decreases (−6.50 mg/dl, 95% CI −9.10 to −3.90; *p* < 0.001, *q* < 0.001), mirror intensive interventions, indicating broader cardioprotective effects mediated by individualized monitoring (22, 24). These cardiovascular improvements are particularly significant given that the leading cause of mortality in older adults with diabetes is cardiovascular disease, suggesting that high-fidelity CGA implementation may be associated with lower long-term morbidity and mortality; prospective studies are needed to test this hypothesis.

Implementation of fidelity explained 18% of variance in HbA1c, indicating it represents one important but not dominant factor in glycemic outcomes. The substantial unexplained variance (82%) likely reflects unmeasured determinants including patient factors (genetics, medication adherence, and lifestyle), environmental factors (food access, social support), healthcare system factors (care continuity, specialist access), and provider factors (communication skills, experience). This aligns with diabetes control's multifactorial nature and suggests high-fidelity CGA should be viewed as a valuable component of comprehensive management rather than standalone solution. Despite explaining minority variance, fidelity's effect size (0.73 percentage point HbA1c improvement) remains clinically meaningful, comparable to adding diabetes medication.

Patient-centered outcomes improved significantly with higher fidelity, encompassing EuroQol-5D index (0.061, 95% CI 0.041–0.081; *p* < 0.001, *q* < 0.001), activities of daily living (0.58, 95% CI 0.39–0.77; *p* < 0.001, *q* < 0.001), and reduced Geriatric Depression Scale scores (−1.10, 95% CI −1.40 to −0.80; *p* < 0.001, *q* < 0.001), aligning with reports of enhanced well being in structured care models (19, 24). These gains likely stem from personalized goal-setting and engagement, mitigating risks of functional decline and mental health issues in diabetic elders (22, 30). The 0.061-point improvement in EQ-5D utility scores exceeds the minimal clinically important difference of 0.05, indicating that patients would perceive meaningful improvements in their overall quality of life. Healthcare utilization decreased markedly, with hospitalization incidence rate ratios of 0.61 (95% CI 0.51–0.73; *p* < 0.001) in highest vs. lowest quartiles, which may have economic implications; however, this study did not perform a formal cost-effectiveness analysis (19, 22). Although the observed reductions in hospitalizations and emergency department visits imply potential resource savings, these findings do not constitute a cost-effectiveness analysis. A rigorous economic evaluation incorporating intervention delivery costs, downstream healthcare expenditures, and health utility was beyond the scope of this study and should be addressed in future work.

Safety outcomes revealed fidelity's association with lower odds of hypoglycemic events (adjusted odds ratio 0.78 per 0.10-unit increase, 95% CI 0.72–0.84; *p* < 0.001), independent of insulin use and comorbidity, consistent with structured protocols reducing adverse risks (19, 24). Effect modification by age (*p* = 0.04) and gait speed (*p* = 0.02) indicated stronger associations in younger seniors (65–74 years) and those with preserved mobility, highlighting functional reserve as a moderator (24, 29). These findings suggest that CGA implementation may be most beneficial when initiated earlier in the aging process, before significant functional decline occurs, supporting preventive rather than reactive care models. Exploratory mediation analysis estimated that functional status accounted for 31.6% of the association between fidelity and HbA1c (natural indirect effect β −0.12, 95% CI −0.17 to −0.07; *p* < 0.001), robust to sensitivity analyses, elucidating pathways linking process quality to metabolic outcomes (19, 24). This mediation analysis provides important mechanistic insights, suggesting that CGA improves glycemic control not only through direct diabetes management but also by enhancing patients' functional capacity for self-care activities.

Sensitivity analyses, including instrumental variable estimation using training completion status (β −0.41, 95% CI −0.54 to −0.28; *p* < 0.001) and complete-case approaches, confirmed robustness (22, 29). Determinants of higher fidelity encompassed patient educational attainment (β 0.067 for high school or higher education, 95% CI 0.041–0.093; *p* < 0.001), provider diabetes experience (β 0.045 per year, 95% CI 0.028–0.062; *p* < 0.001), and CGA training completion (β 0.078, 95% CI 0.048–0.108; *p* < 0.001), explaining 28% of variance and aligning with individual-level implementation readiness literature (19, 22). These factors suggest that targeted interventions focusing on patient education, provider training, and experience enhancement could standardize care and reduce disparities within healthcare institutions (24). Our findings align with the implementation of science frameworks such as the Consolidated Framework for Implementation Research, particularly highlighting the importance of individual characteristics including knowledge, skills, and self-efficacy. The strong association between provider experience and fidelity suggests that supporting professional development is prerequisite for high-quality implementation, with potential training thresholds that healthcare administrators should target to optimize outcomes.

Provider training and experience emerged as particularly important implementation facilitators, suggesting that continuing education and mentorship should be prioritized in CGA scaling efforts. The specific factors that enhanced fidelity included years of diabetes care experience, completion of comprehensive training programs, and patient engagement strategies, providing a blueprint for professional development investments. In comparison with prior nurse-led diabetes interventions, the present findings extend evidence by quantifying fidelity's role in outcomes comparable to multidisciplinary models, advocating CGA as a scalable approach (19, 24, 33, 34). Training program integration enhanced fidelity, consistent with educational studies promoting skill development for precision care (22, 29). Biological mechanisms, including reduced glycemic variability via proactive monitoring, may underpin these associations, with functional improvements driving self-management (24, 30). Implications include institutional prioritization of training and professional development for value-based care, fostering patient engagement and care quality (19, 22).

Based on our findings, healthcare institutions implementing CGA for older adults with diabetes should prioritize several key elements: (1) ensuring comprehensive nurse training programs with minimum 16-h initial training plus ongoing supervision and support, (2) recruiting and retaining experienced diabetes care nurses with demonstrated competency in geriatric assessment, (3) implementing systematic patient education initiatives to improve health literacy and engagement, and (4) establishing quality monitoring systems to track fidelity metrics across providers. Healthcare administrators should recognize that partial implementation may yield suboptimal returns, as our dose-response findings demonstrate incremental benefits across the fidelity spectrum. For clinical practice, our results suggest that CGA should be conceptualized not as an additional assessment but as a systematic approach to reorganizing diabetes care around geriatric principles. Nurses should receive intensive training to integrate functional, cognitive, and social assessments into routine diabetes encounters, with particular attention to individualized goal-setting that accounts for patients' functional capacity and life expectancy. The protective effect against hypoglycemic events suggests that CGA protocols should emphasize safety considerations in medication management, particularly for insulin-treated patients.

Several limitations should be acknowledged. Our fidelity determinant model explained only 28% of variance, indicating 72% of influencing factors remain unmeasured. This includes organizational factors (unit culture, workload, and leadership support), individual nurse factors (self-efficacy, motivation, and communication skills), patient factors (health literacy, family support, and cultural beliefs), and contextual factors (visit timing, seasonal variations, and peer influence). This highlights implementation complexity and suggests successful CGA scaling requires attention to multilevel determinants. Future research should employ mixed methods approaches to identify these unmeasured barriers and facilitators. First, cross-sectional design limits causal inference despite our use of instrumental variable analysis and mediation techniques. Longitudinal studies are needed to establish temporal relationships definitively. Second, while our sample was large and conducted in a major tertiary hospital, all participants were from a single institution in China, potentially limiting generalizability to other healthcare settings and cultural contexts. Third, fidelity assessment, while psychometrically robust, relied partly on self-report measures that may be subject to social desirability bias. Fourth, we did not capture long-term outcomes such as cardiovascular events or mortality, which would strengthen the clinical significance of our findings. Fifth, the study did not include economic evaluation beyond healthcare utilization, limiting assessment of cost-effectiveness. Sixth, unmeasured confounders such as patient motivation or individual provider characteristics beyond those measured may influence both fidelity and outcomes, despite our comprehensive covariate adjustment. Finally, five imputations were used for missing data. Although employing a larger number of imputations can further reduce Monte Carlo error, diagnostic checks supported the sufficiency of *m* = 5 for this dataset. Future research should include randomized trials with long-term follow-up to establish causality, assess cost-effectiveness, and evaluate outcomes such as mortality and quality-adjusted life years. Studies across diverse healthcare institutions and systems are needed to assess generalizability and inform contextual adaptations. Research should also identify optimal training strategies to enhance fidelity while conserving resources. These findings are especially relevant for tertiary care institutions and academic medical centers, where comprehensive nurse training programs and experienced providers support scalable, evidence-based geriatric diabetes care.

## 5 Conclusion

The integration of comprehensive geriatric assessment into routine nursing care for older adults with type 2 diabetes may represent a significant advancement in chronic disease management. High implementation fidelity was strongly associated with multiple clinical benefits, including improved glycemic control, better cardiometabolic profiles, enhanced functional independence, reduced depressive symptoms, and decreased healthcare utilization. These positive outcomes, demonstrated through a clear dose-response relationship and mediated partly through functional status improvements, highlight the critical importance of implementation fidelity in delivering effective chronic disease care. Individual factors such as provider experience, training completion, and patient educational attainment emerged as key determinants of implementation quality, particularly important for optimizing care within healthcare institutions. The association with lower odds of hypoglycemic events and fewer acute-care episodes suggests potential safety and economic implications; however, formal cost-effectiveness was not assessed in this study. The finding that younger seniors and those with better mobility derived greater benefits points toward precision approaches in geriatric diabetes care to maximize intervention effectiveness. Collectively, these results support a fundamental shift toward systematic, high-fidelity nurse-led care models, supported by targeted investments in provider training and patient education, to address the growing burden of diabetes in aging populations and promote equitable, value-based healthcare delivery within healthcare institutions.

## Data Availability

The raw data supporting the conclusions of this article will be made available by the authors, without undue reservation.
