# Supplementary material for: Integrating comprehensive geriatric assessment into routine nursing care for older adults with type 2 diabetes: implementation fidelity and clinical outcomes
Source: Front Public Health. 2025 Sep 19;13:1665732. doi: 10.3389/fpubh.2025.1665732 (PMC12490985; doi:10.3389/fpubh.2025.1665732)
Supplement: Supplementary file 1 [file Table_1.docx]

**Supplementary Table 1:** Baseline Characteristics by Implementation Fidelity Quartiles

| Characteristic | Q1 (n=838) | Q2 (n=838) | Q3 (n=837) | Q4 (n=838) | P value*^1^* |
| --- | --- | --- | --- | --- | --- |
| Sociodemographic characteristics |  |  |  |  |  |
| Age, mean (SD), y | 73.4 (6.9) | 73.2 (6.8) | 73.0 (6.7) | 73.1 (6.8) | 0.663 |
| Female sex, No. (%) | 436 (52.0) | 438 (52.3) | 431 (51.5) | 438 (52.3) | 0.987 |
| Educational attainment, No. (%) |  |  |  |  |  |
| Primary school or less | 502 (59.9) | 456 (54.4) | 426 (50.9) | 406 (48.4) |  |
| Middle school | 256 (30.5) | 264 (31.5) | 267 (31.9) | 265 (31.6) | <0.001 |
| High school or higher | 80 (9.5) | 118 (14.1) | 144 (17.2) | 167 (19.9) |  |
| Household income, No. (%) |  |  |  |  | <0.001 |
| Low | 551 (65.8) | 518 (61.8) | 496 (59.3) | 467 (55.7) |  |
| Middle | 229 (27.3) | 246 (29.4) | 249 (29.8) | 267 (31.9) |  |
| High | 58 (6.9) | 74 (8.8) | 92 (11.0) | 104 (12.4) |  |
| Married, No. (%) | 592 (70.6) | 598 (71.4) | 596 (71.2) | 601 (71.7) | 0.970 |
| Clinical characteristics |  |  |  |  |  |
| Diabetes duration, mean (SD), y | 9.9 (7.3) | 9.8 (7.2) | 9.7 (7.1) | 9.8 (7.2) | 0.956 |
| Insulin therapy, No. (%) | 342 (40.8) | 336 (40.1) | 332 (39.7) | 331 (39.5) | 0.949 |
| BMI, mean (SD), kg/m² | 25.1 (3.8) | 24.8 (3.6) | 24.7 (3.5) | 24.6 (3.4) | 0.027 |
| Charlson Comorbidity Index, mean (SD) | 1.9 (1.9) | 1.8 (1.9) | 1.8 (1.9) | 1.7 (1.8) | 0.190 |
| Provider characteristics |  |  |  |  |  |
| Primary nurse diabetes experience, y | 7.8 (4.1) | 8.2 (4.2) | 8.6 (4.3) | 9.0 (4.2) | <0.001 |
| CGA training completed, No. (%) | 762 (90.9) | 789 (94.2) | 800 (95.6) | 805 (96.1) | <0.001 |

*^1^*P values from ANOVA for continuous variables, χ² test for categorical variables. Abbreviations: BMI, body mass index; CGA, comprehensive geriatric assessment.
